# Management of Treatment-Resistant Depression: An Updated Narrative Review of Current and Emerging Treatments

**DOI:** 10.7759/cureus.107343

**Published:** 2026-04-19

**Authors:** Juan Pablo Berlin Viniegra

**Affiliations:** 1 School of Medicine, Universidad Autónoma de Guadalajara (UAG), Guadalajara, MEX

**Keywords:** augmentation strategies, deep brain stimulation, electroconvulsive therapy, esketamine, ketamine, major depressive disorder, transcranial direct current stimulation, transcranial magnetic stimulation, treatment resistant depression, vagus nerve stimulation

## Abstract

Treatment-resistant depression (TRD) remains a major clinical challenge in psychiatric practice and affects a substantial proportion of patients with major depressive disorder (MDD). It is commonly defined as failure to achieve remission after at least two adequately conducted antidepressant trials. TRD is associated with persistent functional impairment, increased morbidity, reduced quality of life, and greater healthcare utilization. This narrative review summarizes current and emerging evidence on the management of TRD, with emphasis on conventional pharmacological strategies, augmentation approaches, ketamine and esketamine, and neuromodulatory approaches. Neuromodulatory interventions discussed include electroconvulsive therapy (ECT), repetitive transcranial magnetic stimulation (rTMS), and innovative modalities such as theta burst stimulation (TBS), transcranial direct current stimulation (tDCS), vagus nerve stimulation (VNS), and deep brain stimulation (DBS). Emerging therapies, including psychedelic-assisted treatments, are also considered as potential future directions in the management of TRD. While conventional therapies remain central to management, newer treatment modalities have expanded available options, particularly for patients with severe or persistent symptoms. Nevertheless, significant limitations remain, including cost, accessibility, durability of response, and uncertainty regarding long-term outcomes and side effects. This review also highlights the importance of individualized selection and clinical sequencing based on patient characteristics, prior treatment response, and illness severity. Continued research is needed to better define treatment sequencing and improve management strategies for patients with TRD.

## Introduction and background

Major depressive disorder (MDD) remains a common and clinically significant psychiatric disorder in which a substantial proportion of patients do not respond adequately to first-line treatment. Although many patients improve with standard guideline-based treatment, a substantial proportion fail to respond adequately to first-line interventions. Treatment-resistant depression (TRD) is commonly defined as failure to achieve remission after at least two adequately conducted antidepressant trials [1,2].

The clinical importance of TRD extends beyond symptom persistence alone. Patients with TRD often experience greater chronicity of depressive symptoms, more frequent relapse, more pronounced functional decline, and poorer overall outcomes compared with those who respond to initial treatment [2,3]. The condition also carries an important functional and socioeconomic burden through impaired occupational functioning and reduced quality of life [3].

In recent years, multiple reviews have examined individual treatment modalities for TRD; many focus on specific interventions such as pharmacologic augmentation, ketamine-based therapies, or neuromodulation techniques [4]. Given the expanding range of available therapies and the evolving clinical landscape, there remains a need for a concise overview integrating conventional, rapid-acting, and emerging neuromodulatory strategies for the management of TRD within a clinically oriented framework.

Methodology

A targeted literature search was conducted using PubMed and selected major psychiatry journals. Search terms included “treatment-resistant depression”, “major depressive disorder”, “ketamine”, “esketamine”, “transcranial magnetic stimulation”, “electroconvulsive therapy”, “deep brain stimulation”, “transcranial direct-current stimulation”, “vagus nerve stimulation”, and “augmentation strategies”. Articles published between 2000 and 2025 were considered. The initial search yielded approximately 50 records. After removal of duplicates and exclusion of studies not directly relevant to the clinical management of TRD, and non-English publications, approximately 40 articles were reviewed in full. Of these, 26 key articles, studies, and reviews were selected based on clinical relevance, study design, and contribution to the understanding of current and emerging treatment strategies.

## Review

Current standard management

The initial treatment of MDD typically relies on antidepressant pharmacotherapy, most commonly selective serotonin reuptake inhibitors (SSRIs) or serotonin-norepinephrine reuptake inhibitors (SNRIs). When patients do not achieve adequate improvement after an initial antidepressant trial, clinicians usually consider dose optimization, switching to a different antidepressant, or the combination of agents with complementary mechanisms of action [4].

In TRD, augmentation strategies are commonly considered before moving to more specialized interventions. Frequently used augmentation agents include lithium, atypical antipsychotics such as quetiapine or aripiprazole, and thyroid hormone supplementation [4,5]. These approaches can improve response rates in selected patients and remain part of the commonly used treatment strategies [4]. However, their benefits are often modest, and adverse effects such as sedation, weight gain, metabolic complications, and tolerability issues can limit long-term adherence [4]. Certain anticonvulsants like lamotrigine have been explored as augmentation strategies supporting antidepressant effects in controlled augmentation studies [6]; however, their role remains less well established compared to more widely supported approaches.

Psychotherapy also continues to play an important role in the management of depression and depression-related disorders. Cognitive behavioral therapy (CBT), in particular, may improve outcomes when used alongside pharmacotherapy. Still, despite combination treatment, a significant proportion of patients continue to experience persistent depressive symptoms, highlighting the limitations of conventional treatment algorithms and the need for more effective or mechanistically distinct interventions [3].

Innovative and emerging treatments

Ketamine and Esketamine

One of the most important developments in the treatment of TRD has been the introduction of ketamine-based therapies. Ketamine, an N-methyl-D-aspartate (NMDA) receptor antagonist, differs fundamentally from conventional antidepressants by targeting glutamatergic signaling rather than monoamine reuptake. This mechanism differs primarily from conventional monoaminergic antidepressants and may help explain its rapid antidepressant effects [7,8].

Several studies have demonstrated that ketamine can produce clinically meaningful symptom improvement within hours to days, a timeline that contrasts with the delayed onset seen with traditional antidepressants [7,8]. This rapid onset is especially relevant in acute clinical settings, such as severe depressive episodes with suicidal ideation or marked functional deterioration.

Intranasal esketamine has emerged as a standardized formulation for use in TRD and has received regulatory approval in specific clinical settings [9,10]. Compared with intravenous ketamine, intranasal esketamine offers a standardized route of administration and has become increasingly integrated into clinical practice [10]. However, despite its promise, several practical and clinical limitations remain.

First, the antidepressant effects of ketamine-based interventions may not be durable without repeated administration. Many patients experience relapse after an initial response, raising questions regarding maintenance plans and long-term treatment outcomes [8,9]. Second, although these therapies may be highly effective for some individuals, they are often expensive and not uniformly accessible [11]. Finally, dissociative symptoms, transient increases in blood pressure, and the need for supervised administration can complicate treatment delivery [7,11].

In practical terms, ketamine and esketamine should be viewed less as replacements for standard antidepressants and more as important additions for carefully selected patients. Their greatest value may lie in situations where rapid symptom relief is especially important or where conventional strategies have repeatedly failed [7,10,12,13].

Repetitive Transcranial Magnetic Stimulation (rTMS)

rTMS has become an increasingly important non-invasive treatment option for patients with TRD. rTMS uses electromagnetic stimulation directed to cortical regions implicated in mood regulation, most commonly the dorsolateral prefrontal cortex. Unlike pharmacological interventions, it does not rely on systemic drug exposure, making it particularly attractive for patients who are unable to tolerate medication side effects [14-16].

Clinical trials and meta-analyses have demonstrated that rTMS can provide meaningful symptom reduction in patients who have not responded to antidepressant therapy [14-17]. Its safety profile is generally favorable, and most adverse effects are relatively mild, including headache, scalp discomfort, or transient fatigue [14-17]. The risk of serious complications, such as seizure, is low when proper protocols are followed [15,17].

Despite these advantages, rTMS is not exempt from limitations. Response rates are variable, and treatment often requires multiple sessions over several weeks, which can reduce convenience and limit adherence [14,17]. Accessibility also remains a practical barrier in many regions due to cost, equipment availability, and the need for specialized treatment centers [14].

When compared with ketamine-based treatments, rTMS generally has a slower onset of action but may be preferable in patients seeking a non-pharmacologic option with fewer systemic adverse effects. In practice, the choice between rTMS and ketamine often depends on clinical urgency, patient preference, cost, comorbidities, and local availability [11,14-17].

Electroconvulsive Therapy (ECT)

ECT remains one of the most effective interventions for severe and refractory depression. Although often reserved for advanced or urgent cases, it continues to hold a central place in the management of TRD, particularly in patients with psychotic depression, catatonia, marked suicidality, severe functional decline, or failure of multiple prior treatment modalities [18,19].

ECT demonstrates high response and remission rates and remains one of the most effective available interventions for severe depression [18]. For this reason, it remains a treatment of choice in several severe presentations of TRD. In practice, it may be especially valuable when a rapid and robust response is required [19].

Nevertheless, ECT remains underutilized. Much of this underuse is related not to poor efficacy, but to persistent stigma, patient apprehension, misconceptions regarding safety, and concerns about cognitive side effects [18,19]. Memory impairment, particularly around the time of treatment, remains one of the most commonly cited barriers. While cognitive effects are often transient and may be outweighed by the severity of untreated illness, they remain clinically relevant and should be discussed openly with patients [19].

Compared with newer therapies such as ketamine or rTMS, ECT may appear more invasive, but it remains the most consistently effective intervention for severe TRD. In that sense, its role has not been displaced by emerging treatments, but rather complemented by them [18,19].

Deep Brain Stimulation (DBS)

DBS represents a more invasive but conceptually important tool in the treatment of TRD. DBS involves surgical implantation of electrodes into specific neural circuits implicated in mood regulation. The rationale for this approach reflects a broader shift in the understanding of depression as a disorder of dysfunctional neural networks rather than exclusively a disturbance of neurotransmitter availability [20,21].

Although DBS has shown promise in selected studies, its results remain mixed, and its clinical use is currently limited primarily to research or highly specialized settings [20,21]. Variability in outcomes, uncertainty regarding optimal targets, and the invasive nature of the intervention have limited widespread adoption [20,21].

Other Neuromodulation Techniques

Additional neuromodulation techniques have also been explored. Theta burst stimulation (TBS), a form of transcranial magnetic stimulation that implements higher frequency pulses in rapid bursts, has demonstrated non-inferiority to conventional rTMS protocols while significantly reducing treatment duration [22].

Transcranial direct current stimulation (tDCS) represents another non-invasive approach, although existing evidence suggests more modest and less consistent effects compared to established modalities [23].

Vagus nerve stimulation (VNS), an invasive neuromodulation technique, is a known intervention in chronic or treatment-resistant depression in selected patients, although its clinical use remains limited by cost, invasiveness, and delayed therapeutic onset [24].

Emerging Therapies

A growing number of emerging therapies continue to reshape the treatment landscape of TRD. These include newer glutamatergic approaches, individualized neuromodulation strategies, and interventions aiming at resistant symptom domains more directly [11,20,21]. Together, these approaches reflect the clinical reality that TRD is heterogeneous and may require more individualized treatment strategies [3,11].

This evolving framework may ultimately support more personalized treatment selection. However, current evidence still falls short of providing definitive sequencing strategies for many of these interventions, and long-term comparative data remain limited.

Psychedelic-assisted therapies, including psilocybin, have recently gained attention as potential treatments for TRD. Early studies suggest rapid and sustained antidepressant effects in controlled settings when combined with psychological support [25]. However, these approaches remain investigational, and further research is required to establish long-term safety and clinical applicability [26].

Treatment considerations and clinical sequencing

In clinical practice, the management of treatment-resistant depression requires an individualized and stepwise approach. After failure of initial antidepressant trials, clinicians typically consider dose optimization, switching antidepressants, or pharmacologic augmentation.

When these strategies are insufficient, non-pharmacologic interventions such as rTMS or ketamine-based therapies may be considered depending on clinical urgency, patient preference, safety profile, and availability.

Electroconvulsive therapy remains the most effective option for severe or refractory cases, particularly in patients with psychotic features, severe functional impairment, or acute suicidality.

Emerging approaches may play a role in particular patients, although their use remains limited. Treatment decisions should be guided by clinical severity, prior response, comorbidities, safety profile, and accessibility.

## Conclusions

TRD remains a major clinical challenge and continues to require a comprehensive and individualized approach to management. Conventional strategies such as antidepressant switching, pharmacologic augmentation, and psychotherapy remain foundational, but they are often insufficient for many patients with persistent or severe illness.

Recent advances have significantly expanded available treatment options. Ketamine and esketamine have introduced a rapid-acting approach with clear clinical relevance, particularly in acute or refractory cases. rTMS offers a non-invasive neuromodulatory option with good tolerability, while ECT remains the most effective intervention for severe and urgent presentations. Emerging approaches such as DBS may further broaden future treatment possibilities, although more evidence is still needed before routine clinical adoption. Overall, the treatment of TRD increasingly requires a more individualized and mechanism-informed approach to care. More research is still needed to clarify long-term efficacy and safety to define treatment suitability and to improve outcomes for this difficult-to-treat group.
